# *In vitro* evaluation of AI-assisted CBCT analysis for detecting additional canals in mandibular premolars

**DOI:** 10.6026/973206300214922

**Published:** 2025-12-15

**Authors:** Jasmine Marwaha, Nehali Thakkar, Bassam Alkhalifah, Arindam Banik, Nilesh Dinesh Pardhe, Mohammed Mustafa

**Affiliations:** 1 Department of Conservative Dentistry and Endodontics, National Dental College and Hospital, Mohali-140507, India; 2 Department of Conservative Dentistry and Endodontics, Pacific Dental College and Research Centre, Udaipur, Rajasthan, India; 3 Department of Radiology, College of Medicine, Qassim University, Buraydah, Saudi Arabia; 4 Department of Conservative Dentistry and Endodontics, Guru Nanak Institute of Dental Sciences and Research, Kolkata, West Bengal, India; 5 Department of Oral Pathology, ESIC Dental College & Hospital, Kalaburagi, Karnataka, India; 6 Department of Conservative Dental Sciences, College of Dentistry, Prince Sattam Bin Abdulaziz University, Al-Kharj 11942, Saudi Arabia

**Keywords:** Artificial intelligence, cone-beam computed tomography, mandibular premolars, root canal anatomy, deep learning, endodontics, micro-CT

## Abstract

Failure to detect additional canals in mandibular premolars can compromise endodontic treatment outcomes. Hence, this *in vitro* study compared an artificial intelligence-assisted CBCT analysis system with endodontic specialists for detecting additional canals, using micro-CT as the gold standard. One hundred and fifty mandibular premolars were analyzed. The AI system demonstrated higher sensitivity (86.8%) and accuracy (90.0%) than specialists, with comparable specificity and interpretation time of less than three seconds per case. Thus, AI-based CBCT analysis appears to be a rapid and reliable adjunct for improved canal detection and preoperative endodontic planning.

## Background:

A thorough knowledge of root canal anatomy is one of the key conditions of successful endodontic therapy, since unnoticed and untreated canals can be considered one of the key reasons of treatment failures [1]. The mandibular premolars are especially problematic because they are highly morphologically variable, with other canals also being reported to occur in 12-45 percent of most populations [2]. These are variations that comprise a variety of canals, bifurcations of canals, lateral canals and intricate anastomoses that could go undetected from a standard clinical examination [3]. Cone-beam computed tomography (CBCT) has transformed the process of endodontic diagnosis and teaching planning through the three-dimensional visualization of the dental anatomy with high detail than the conventional radiography [4]. CBCT allows non-invasive evaluation of canal shapes, detection of other canals as well as anatomical complexities that affect treatment management [5]. Nevertheless, CBCT interpretation is a rather complex task and the results of diagnosis depend on the experience of the observer, observation schemes and the complexity of the anatomical structures themselves [6]. The latest advances in artificial intelligence (AI), especially the deep learning algorithms based on convolutional neural networks (CNNs), have revealed impressive opportunities in the field of medical image analysis in a variety of applications [7]. AI systems have been demonstrated to be useful in dentistry, with automated anatomical structure detection, pathology and treatment planning support [8]. AI usage to analyze CBCT in endodontic is a newer sphere, which can increase the quality of diagnoses and save time on interpretation [9]. A number of pre-investigations in this field have examined the use of AI in root canal detection and morphology classification [10]. These works have shown promising findings, where AI systems were able to detect canal orifices, follow canal routes and classify canal outfits based on well-established classification frameworks [11]. Nevertheless, AI performance in identifying a farther canal in mandibular premolars as a task of clinical importance due to the high occurrence of anatomical differences has little literature in specific assessment [12]. Micro-computed tomography (micro-CT) is a high-resolution 3D imaging that is popularly accepted as the gold standard of assessing the anatomy of root canals *ex vivo* [13]. Micro-CT may be used to determine canal systems with a resolution that is above the clinical CBCT technique, which is the best reference standard to test the diagnostic tools [14]. Although there is an increased enthusiasm in AI usage in endodontic imaging, there are numerous gaps in the knowledge about the comparative diagnostic efficiency of AI systems and skilled endodontists in identifying additional canals in complex teeth anatomy, like mandibular premolars. Moreover, there are not many data available on the kind of canal arrangements that artificial intelligence detectors find the most difficult and the ideal ways to introduce AI in clinical operations [15]. Therefore, it is of interest to assess the diagnostic accuracy, sensitivity and specificity of a CNN-based AI system relative to board-certified endodontic specialists in identifying additional canals in mandibular premolars with CBCT imaging with micro-CT as a reference standard.

## Materials and Methods:

## Sample size calculation:

Based on preliminary data and assuming AI sensitivity of 85% versus specialist sensitivity of 75%, with α=0.05 and power=0.80, a minimum of 132 specimens was required. Accounting for potential technical failures, 150 mandibular premolars were included.

## Inclusion criteria:

Extracted human mandibular first and second premolars; mature roots with closed apices; intact root structure without fractures or resorption; absence of previous endodontic treatment; no evidence of calcification obscuring canal anatomy; roots longer than 15mm.

## Exclusion criteria:

Teeth with open apices or developmental anomalies; presence of posts, restorations or materials within the canal space; visible external or internal resorption; fractures or cracks visible under magnification; severe calcification; incomplete root formation. One hundred fifty mandibular premolars (87 first premolars, 63 second premolars) meeting inclusion criteria were selected from the biobank. Teeth were cleaned of soft tissue and calculus using ultrasonic scaling, stored in 0.1% thymol solution at 4°C and allowed to reach room temperature before imaging.

## Micro-CT Imaging (Reference Standard):

All specimens underwent micro-CT scanning (SkyScan 1272, Bruker, Belgium) using the following parameters: voltage 80kV, current 125µA, rotation step 0.3°, frame averaging 3, voxel size 20µm, 360° rotation. Specimens were secured in custom holders to prevent movement during scanning. Micro-CT data were reconstructed using NRecon software (Bruker) with beam hardening correction (30%), ring artifact reduction (8) and consistent threshold values. Three-dimensional models were generated using CTVox software (Bruker).

Two experienced endodontists with micro-CT expertise independently analyzed all specimens to establish the reference standard, documenting:

[1] Total number of canals

[2] Presence and location of additional canals

[3] Canal configuration according to Vertucci classification

[4] Bifurcation and anastomosis patterns

Cases with disagreement underwent collaborative review until consensus was achieved. The reference standard diagnosis was established before CBCT evaluation and AI testing.

## CBCT imaging:

Specimens were embedded in pink wax within a dry human mandible to simulate clinical conditions and scatter radiation patterns. CBCT scans were acquired using a clinical scanner (ProMax 3D Mid, Planmeca, Finland) with standardized parameters: field of view 8x8cm, voxel size 0.1mm, 90kV, 12mA, exposure time 12 seconds. Each tooth was scanned individually with consistent positioning. CBCT data were exported in DICOM format and anonymized. All identifying information was removed and specimens were assigned random numerical codes.

## AI system development and architecture:

A custom CNN-based AI system was developed using Python 3.9 with PyTorch 1.12 framework. The system architecture comprised:

## Training dataset:

The AI network was trained on 2,500 CBCT scans of mandibular premolars (separate from the 150-specimen test set) collected from multiple institutions, with expert-annotated canal configurations verified by micro-CT or clearing technique.

## Network architecture:

The model utilized a modified 3D U-Net architecture optimized for volumetric medical image segmentation. The encoder pathway consisted of five levels with 3D convolutional layers (kernel size 3x3x3), batch normalization, ReLU activation and max pooling. The decoder pathway employed transposed convolutions for upsampling with skip connections from corresponding encoder levels. The final layer used softmax activation for multi-class segmentation (background, dentin, canal space).

## Data preprocessing:

CBCT volumes were resampled to isotropic 0.1mm voxels, normalized to 0-1 intensity range and augmented during training using random rotations (±15°), scaling (0.9-1.1), elastic deformations and Gaussian noise addition.

## Training protocol:

The model was trained for 200 epochs using AdamW optimizer (learning rate 0.001 with cosine annealing), dice coefficient loss function combined with focal loss and batch size of 4. Training utilized NVIDIA RTX A6000 GPU. Validation set (500 scans) monitored performance, with early stopping if validation loss did not improve for 20 epochs.

## Output and post-processing:

For each test specimen, the AI system generated:

[1] 3D segmentation of canal space

[2] Automated canal counting and configuration classification

[3] Confidence score for detection (0-1 scale)

[4] Color-coded visualization highlighting detected canals

[5] Processing time log

Post-processing algorithms applied morphological operations (erosion-dilation) to remove noise and connected component analysis to identify discrete canal structures.

## Endodontist evaluation:

Five board-certified endodontists (mean experience: 14.2 ± 5.8 years post-certification) independently evaluated all 150 CBCT scans. Evaluators were blinded to micro-CT findings, AI results and each other's assessments. CBCT volumes were viewed using dedicated viewing software (Romexis Viewer, Planmeca) on calibrated 27-inch monitors (2560x1440 resolution) in darkened rooms. Evaluators could freely navigate through axial, coronal and sagittal planes; adjust window/level settings; create curved multiplanar reconstructions; and use measurement tools. No time restrictions were imposed.

For each specimen, endodontists documented:

[1] Total number of canals detected

[2] Presence and location of additional canals (beyond single canal)

[3] Canal configuration classification (Vertucci Type I-VIII or additional categories)

[4] Confidence level (1-5 scale: 1=very uncertain, 5=very certain)

[5] Evaluation time

Evaluations were conducted across multiple sessions (maximum 30 specimens per session) separated by minimum 48 hours to minimize fatigue.

## Outcome measures:

## Primary outcomes:

[1] Sensitivity for detecting additional canals

[2] Specificity for correctly identifying single-canal anatomy

[3] Overall diagnostic accuracy

[4] Positive predictive value (PPV)

[5] Negative predictive value (NPV)

## Secondary outcomes:

[1] Canal configuration classification accuracy

[2] Detection rate by configuration type

[3] Inter-rater reliability among specialists

[4] Agreement between AI and micro-CT reference standard

[5] Processing/evaluation time

[6] Confidence level correlation with accuracy

## Definitions:

[1] Additional canal: presence of ≥2 canals at any root level confirmed by micro-CT

[2] True positive: correctly detecting additional canal(s) when present

[3] True negative: correctly identifying single canal when only one exists

[4] False positive: detecting additional canal(s) when single canal present

[5] False negative: failing to detect additional canal(s) when present

## Statistical analysis:

Statistical analyses were performed using SPSS version 28.0 (IBM Corp.) and R version 4.3.0 (R Foundation). Descriptive statistics included mean ± standard deviation for continuous variables and frequencies (percentages) for categorical variables. Sensitivity, specificity, accuracy, PPV and NPV were calculated with 95% confidence intervals using the Clopper-Pearson method. Comparisons between AI and mean specialist performance utilized McNemar's test for paired proportions and independent t-tests for continuous metrics. Inter-rater reliability among the five specialists was assessed using Fleiss' kappa. Agreement between AI and reference standard and between individual specialists and reference standard, was evaluated using Cohen's kappa coefficient. Kappa interpretation followed standard criteria: <0.20=slight, 0.21-0.40=fair, 0.41-0.60=moderate, 0.61-0.80=substantial, 0.81-1.00=almost perfect. Chi-square tests compared categorical outcomes (canal configuration classification accuracy). Repeated measures ANOVA evaluated differences across multiple evaluators with post-hoc pairwise comparisons using Bonferroni correction. Pearson correlation assessed relationship between confidence levels and accuracy. Subgroup analyses examined performance stratified by tooth type (first versus second premolar), canal configuration type and presence of bifurcations. Statistical significance was set at p<0.05 (two-tailed).

## Results:

The final dataset comprised 150 mandibular premolars (87 first premolars, 58.0%; 63 second premolars, 42.0%). Micro-CT reference standard analysis revealed additional canals in 68 specimens (45.3%) and single-canal anatomy in 82 specimens (54.7%). Canal configuration distribution according to micro-CT: Vertucci Type I (single canal) 54.7% (n=82), Type II (2-1 configuration) 12.0% (n=18), Type III (1-2-1 configuration) 8.7% (n=13), Type IV (two separate canals) 10.0% (n=15), Type V (1-2 configuration) 9.3% (n=14), Type VI and other complex configurations 5.3% (n=8). Additional canals were more prevalent in first premolars (52.9%, 46/87) compared to second premolars (34.9%, 22/63), p=0.028. Mean root length was 21.4 ± 2.8mm. Table 1 (see PDF) presents comprehensive diagnostic performance metrics for AI system and individual endodontic specialists. The AI system demonstrated significantly higher sensitivity (86.8%) compared to mean specialist sensitivity (79.4%, p=0.021). Specificity was comparable between AI (92.7%) and specialists (89.5%, p=0.187). Overall accuracy was significantly higher for AI (90.0%) versus mean specialists (85.1%, p=0.048). Agreement with micro-CT reference standard was substantial for AI (κ=0.792) and moderately substantial for specialists (mean κ=0.697, p=0.038). False negative rates (failing to detect additional canals when present) were 13.2% (9/68) for AI compared to mean 20.6% for specialists. False positive rates (detecting additional canals when single canal present) were 7.3% (6/82) for AI versus mean 10.5% for specialists. Table 2 (see PDF) presents canal configuration classification accuracy and processing time metrics. AI demonstrated significantly higher overall canal configuration classification accuracy (88.7%) compared to mean specialist performance (83.3%, p=0.038). This advantage was particularly evident for complex multi-canal configurations (Types II-VIII), where AI achieved 80.9% accuracy versus 72.1% for specialists (p=0.019). Processing time differed dramatically, with AI requiring 2.3 ± 0.4 seconds per case compared to 124.7 ± 28.5 seconds for specialists (p<0.001), representing approximately 54-fold faster analysis. Table 3 (see PDF) presents diagnostic performance stratified by tooth type and anatomical complexity. AI demonstrated superior sensitivity to specialists across most subgroups, achieving statistical significance in first premolars (84.8% vs 77.9%, p=0.048). Performance declined for both AI and specialists with increasing anatomical complexity, though AI maintained advantage in complex cases (62.5% vs 56.3%, p=0.509, not significant due to small sample size). Inter-specialist agreement for detecting additional canals demonstrated substantial reliability (Fleiss' κ=0.724, 95% CI: 0.693-0.755). Mean pairwise Cohen's kappa among specialists was 0.738 ± 0.096. Agreement between AI and individual specialists ranged from κ=0.718 to κ=0.766 (mean: 0.744 ± 0.019), comparable to inter-specialist agreement. Correlation between AI confidence scores and diagnostic accuracy was strong (r=0.831, p<0.001). Similarly, specialist confidence levels correlated with accuracy (mean r=0.798 ± 0.087, p<0.001).

## Discussion:

This extensive *in vitro* experiment shows a CNN-based AI system had much better sensitivity and had similar specificity to endodontic board-certified specialists in identifying additional canals in mandibular premolars through CBCT imaging and offered a much lower analysis time. The sensitivity of 86.8% of the AI system is a 7.4 percentage point increase in sensitivity in comparison to the mean specialist sensitivity (79.4%), which may translate to identifying previously missed canals that may undermine treatment outcomes without treatment [1]. The level of diagnosis that was found in this study is consistent with and builds on other studies of AI usage in endodontic imaging. Research done previously indicated AI sensitivity of 72 to 91 on canal detection tasks, even though most were performed under less difficult detection conditions or on a variety of tooth types [2]. Our findings indicate that advanced 3D CNN models can be used successfully to process volumetric CBCT volumes to detect more canals in anatomically varied mandibular premolars, which is clinically important since most of them contain missed canals [3]. An unusual feature of AI that deserves attention is the fact that it is more sensitive than specialists, which implies that deep learning algorithms are able to pick up minor anatomical peculiarities that a human eye can miss. This benefit is probably due to the fact that AI can process the overall volumetric data sets in a systematic manner with no perceptual exhaustion, use uniform decision criteria and identify intricate three-dimensional patterns trained on thousands of training images [4]. The specificity is slightly less than the sensitivity, which means that there is a propensity to false positive detectives, but the 92.7% specificity is a clinically acceptable result and it is in the range of specialist results [5]. Similar trends were seen in accuracy to classify canal configuration with AI doing better than specialists with complex multi-canal configuration (Types II-VIII). This observation is indicative that AI systems are good at differentiating faint anatomical differences, which define various Vertucci classifications and this may be useful in entire treatment planning beyond merely additional canal identification [6]. The lower precision of very complicated configurations (62.5) is intrinsic to the ability to solve more complex anastomoses and lateral canals being near the resolution boundaries of clinical CBCT [7]. The 54-fold processing time (2.3 seconds versus 124.7 seconds) is an efficiency change that is transformative and has a big clinical workflow impact [8]. This time savings may save a significant amount of time even in endodontic specialty referral centers or in busy endodontic practice where it may result in much productivity increase with no loss or even improvement in diagnostic accuracy. Nevertheless, quick processing must be used to supplement instead of to substitute careful clinical combination of the visual findings with the symptoms of the patients, clinical examination and treatment goals [9]. The stratification of the performance by the type of tooth showed that further canals in first premolars that displayed a greater anatomical variation (52.9% prevalence) could be more effectively observed by AI than by specialists. This result has some specific clinical significance, because first premolars are often taken to endodontic treatment and the anatomical complexity is a direct determinant of the difficulty of treatment and prognosis [10]. The preserved AI difference between the different root lengths and level of anatomy indicates that it has a strong ability to generalize. The inter-rater reliability analysis showed that there is also a significant consensus between specialists (0.724), which imply that specialists do not have a strong agreement, though there is still some important diagnostic variance. The similarity between the agreement of AI and experts (mean κ=0.744) and inter-specialist agreement is an indication that AI also brings about a diagnostic variation that is comparable to that of another expert-specialist, but not systematic bias [11]. The correlation between confidence scores and accuracy between AI and specialists (r>0.79) promotes possible confidence-based workflow integration, whereby large confidence AI judgments are accepted faster than uncertain instances are further subjected to specialist inspection [12]. A number of clinical implications arise out of these findings. To begin with, AI-based CBCT interpretation may be an effective screening device in general dentistry, alerting the presence of likely extra canals to refer to specialists or improve the treatment planning. Second, AI may be a second reader system in place of a human endodontist that checks canal morphology systematically prior to beginning treatment. Third, AI-based applications have educational applications, assisting residents and practitioners in the recognition of complex anatomical variations with the interactive feedback [13]. Micro-CT as the reference standard is also a strength of the research methodology because it offers ultra-high resolution ground truth that is superior to other verification methods. The minor differences between clinical CBCT (100um resolution) and micro-CT (20um resolution) however, create inherent detection limits to both the AI and the human observers to extremely small canals when they are near voxel sizes. Such a limitation is common to all diagnostic methods based on CBCT and can support the significance of using several diagnostic modalities [14]. There are significant restrictions to which it should be recognized. To start with, the study was an *in vitro* study that tested extracted teeth under controlled conditions; in the absence of the clinical nature of patient symptoms, pulp testing outcomes and treatment history that guides diagnostic decisions in actual clinical practice. Second, the AI system was trained using multiple-institutions large dataset but tested using single-center sample which could be enhanced with external validation using various populations and CBCT scanners. Third, the researchers were interested in mandibular premolars and the performance could be different in other types of teeth with different anatomy. Fourth, binary outcome (additional canals present/absent) fails to reflect clinical subtleties (accessibility, negotiability and relevance of very fine lateral canals of treatment) of further canals. Fifth, the CBCT all scans were obtained under the similar protocols of high quality; performance may not be equal when irregular image quality in practice occurs. There are a number of priorities, which should be covered in future researches. Good clinical trials to assess the effects of AI on clinical practice decisions, efficiency of the procedures, or patient outcomes are needed to prove clinical utility. Applicability would be expanded by extension to other types of teeth and creating holistic AIs with the ability to analyze multiple teeth simultaneously. Combination of AI and intraoperative technology like operating microscopy and ultrasonic exploration might establish synergistic diagnostic platforms. Future studies on AI performance in identifying other anatomical findings (isthmuses, lateral canals, apical deltas) and pathology would increase functional abilities. Lastly, the implementation plans would be informed by the cost-effectiveness analysis and workflow integration research [15].

## Conclusion:

The AI-based CBCT analysis system demonstrated higher diagnostic sensitivity and comparable specificity to endodontic specialists while significantly reducing evaluation time. Its strong agreement with micro-CT confirms reliability in detecting complex canal anatomies. Thus, AI-assisted imaging serves as an effective adjunct to enhance endodontic diagnosis and treatment planning when integrated with clinical expertise.
